# Comparison of Two Base Materials Regarding Their Effect on Root Canal Treatment Success in Primary Molars with Furcation Lesions

**DOI:** 10.1155/2016/1429286

**Published:** 2016-11-10

**Authors:** Volkan Arikan, Hayriye Sonmez, Saziye Sari

**Affiliations:** ^1^Faculty of Dentistry, Department of Pedodontics, Kirikkale University, Kirikkale, Turkey; ^2^Faculty of Dentistry, Department of Pedodontics, Ankara University, Ankara, Turkey

## Abstract

*Introduction.* The aim of this study was to compare MTA with another base material, IRM, which is generally used on pulpal floor after root canal treatment, regarding their effect on the success of root canal treatment of primary teeth with furcation lesions.* Materials and Methods.* Fifty primary teeth with furcation lesions were divided into 2 groups. Following root canal treatment, the pulpal floor was coated with MTA in the experimental group and with IRM in the control group. Teeth were followed up considering clinical (pain, pathological mobility, tenderness to percussion and palpation, and any soft tissue pathology and sinus tract) and radiographical (pathological root resorption, reduced size or healing of existing lesion, and absence of new lesions at the interradicular or periapical area) criteria for 18 months. For the statistical analysis, Fisher's exact test and Pearson's chi-square tests were used and a *p* value of <0.05 was considered to be statistically significant.* Results.* Although there were no statistically significant differences between two groups in terms of treatment success, lesions healed significantly faster in the MTA group.* Conclusion.* In primary teeth with furcation lesions, usage of MTA on the pulpal floor following root canal treatment can be a better alternative since it induced faster healing.

## 1. Introduction

The aim of root canal treatment in primary teeth, similar to permanent teeth, is to remove all bacteria; necrotic and vital pulp remnants; and infected dentin and to fill the root canal system hermetically [1, 2]. However, the furcation area in primary teeth and permanent teeth exhibits certain different characteristics. The thin pulpal floor, higher prevalence of accessory canals, and wider dentin tubules found in primary teeth result with high permeability of pulpal floor and [3–7]; these factors are generally considered to cause furcation lesions in primary teeth with irreversible pulpitis or necrotic pulps. Moreover, the permeability of the pulpal floor may increase following lesion formation, since Da Silva et al. [8] reported that there were resorption areas on the cement tissue of the teeth with necrotic pulps and radiographic pathology. Thus, it can be assumed that after lesion formation, the permeability on the pulpal floor may also increase due to pathological changes. Mani et al. [9] reported that, in some primary molar teeth having pulpectomy treatment, Ca(OH)_2_ resorbed only at the coronal part of the root canal and the authors attributed that result to the accessory canals that are present in primary teeth. Therefore, despite careful selection of cases and correct treatment procedures, the communication between pulp and periodontal tissue can cause treatment failure since the connection between pulpal space and furcation area may result with transition of microorganisms and their products [10]. Also, leakage of tissue fluids may provide nutrients for any remaining microorganisms in the root canal system and prevent the healing of inflammation. Therefore the material used to coat pulpal floor following root canal treatment may be in a key role in the prognosis of primary teeth with furcation lesions and eliminating the leakage of inflammatory fluids by sealing the pulpal floor properly following root canal filling may increase the success rate of the endodontic treatment of primary teeth with furcation lesions.

Intermediate restorative material (IRM), which is a reinforced zinc oxide eugenol, is used widely for the dressing of the pulpal floor following root canal filling in primary teeth [11–14]. Although IRM was reported to have good sealing ability, studies comparing this material with mineral trioxide aggregate (MTA) reported that MTA has superior sealing ability when compared to IRM [15, 16]. MTA is a biocompatible material, which was proven to have a great sealing ability and to stimulate healing in hard tissues, and it was shown to be successful in the sealing of root and furcation perforations in many studies [17–20].

Considering the high prevalence of furcation lesions in primary teeth, the number of studies investigating the outcome of treatment in these cases is insufficient and in light of above mentioned data the aim of the present study was to compare MTA with another base material, IRM, which is a commonly used material for the coating of pulpal floor following root canal treatment, regarding their effect on the treatment success in primary teeth with furcation lesions.

## 2. Materials and Methods

### 2.1. Study Population

The study population consisted of 50 children (22 boys and 28 girls) aged 4–9 years each having a mandibular primary molar tooth with a lesion in the furcation area. All children were healthy and cooperative. Ethical approval was received from the Institutional Review Board (PN: 135/2), and informed consent was obtained from participants and their parents. Teeth were included in the study if (i) they had a lesion in the furcation area that did not extend beyond the apical half of the space between bifurcation and permanent tooth germ; (ii) there was no internal/external resorption or inadequate bone support; (iii) the lesion did not involve the crypt of the succedaneous tooth germ; (iv) restoration with a stainless steel crown was possible; and (v) physiological root resorption did not exceed one-third of the root. In order to simplify radiographic examination during diagnosis and follow-up, all teeth included were mandibular primary molars.

### 2.2. Study Groups

The sample size was calculated to be 50 teeth (25 for each group) with a 0.4 effect size, a level of 95% confidence, and power of 80%. Teeth were randomly distributed between the two groups by assigning each tooth a number and then assigning all odd-numbered teeth to the IRM (Dentsply, USA) group (*n* = 25) and all even-numbered teeth to the MTA (Proroot MTA, Dentsply, USA) group (*n* = 25). All treatment procedures were performed by the same pediatric dentist.

### 2.3. Technique

Two-visit pulpectomy was performed for all teeth. A preoperative radiograph was taken at the first visit. Following local anesthesia with Ultracain D-S (Aventis, İstanbul, Türkiye) teeth were isolated. After the removal of all carious structure and accessing the pulp chamber, coronal pulp was excavated, barbed broaches were used to extirpate pulp tissue, and root canals were irrigated using 2.5% sodium hypochlorite (NaOCl). Canal length was determined by taking a radiograph with a fine reamer inserted gently into the canal. The working length was maintained 2 mm short of the apex. Canals were prepared to a master file size of 35 using H-files (G-Star Medical Co. Ltd., Guangdong, China) with a pull-back action. Canals were irrigated with 2 mL of 2.5% NaOCl between each change of instrument. After the preparation was complete, root canals were finally irrigated with NaOCl and physiological saline and dried with paper points and Cresophene (Cresophene, Septodont Ltd., UK) was applied in the pulp chamber with a cotton pellet and tooth was filled with a temporary filling material (Cavit G, 3M ESPE, Germany). After 48 hours, canals were irrigated with NaOCl and physiologic saline, dried with paper points, and filled with a Ca(OH)_2_/iodoform paste (Tg-pex, Technical and General Ltd., UK) using plastic syringe provided by the manufacturer and lentulo spirals. Following root canal fillings, base materials were applied to the cavity floor and cavities were temporarily filled with IRM. For the MTA group, after approximately 3 mm of MTA was placed on the pulpal floor a moistened cotton pellet in contact to MTA was left in the cavity before the application of the temporary filling material. A final radiograph was taken to check the level of root canal filling, which was recorded as either “flush-filled” (filling ended between apex and working length for at least 2 root canals), “underfilled” (filling ended shorter than working length for at least 2 root canals), or “overfilled” (filling extended beyond apex in any of the root canals). After 24 hours, for Group II, temporary filling and moistened cotton pellet were removed and the cavity was filled with metal-reinforced glass ionomer cement (Ketac Molar, 3M ESPE, Germany). For Group I, to achieve standardization, IRM was removed from the cavity until approximately 3 mm of the material is left on the pulpal floor and the cavity was filled with metal-reinforced glass ionomer cement. All teeth were restored with stainless steel crowns (3M ESPE Unitek, USA).

### 2.4. Clinical and Radiographic Examinations

Teeth were followed up clinically and radiographically for 18 months. Follow-up visits were conducted once a month for 3 months, at the 6th month, and once every 6 months for an additional 12 months and two calibrated pediatric dentists performed clinical and radiographic examinations. Examiners were blinded to the groups. Two training sessions were performed for calibration on the follow-up radiographs of 10 primary molar teeth that were not included in the study for the furcal/periapical lesions and external pathological root resorption. Kappa scores for each variable ranged between 0.8 and 1, indicating good agreement in the second session.

Treatment success was evaluated based on the following clinical and radiographic criteria:

- Absence of pain.
- Absence of pathological mobility.
- Absence of tenderness to percussion and palpation.
- Absence of any soft tissue pathology and sinus tract.
- Absence of pathological root resorption.
- Reduced size or healing of existing lesion.
- Absence of new lesions at the interradicular or periapical area.

### 2.5. Statistical Analysis

Statistical analysis was performed using the software program SPSS 20.0 (SPSS 20.0 for Windows; SPSS Inc., Chicago, IL, USA). Fisher's exact test was used to compare success rates of the groups and to evaluate the effect of filling extent to success rates. To compare two groups regarding healing time, Fisher's exact test and Pearson's chi-square tests were used and a *p* value of <0.05 was considered to be statistically significant.

## 3. Results

A total of 50 teeth were treated and followed up clinically and radiographically for 18 months.

The root canal fillings were considered as flush for 15 teeth and as overfilled for 20 teeth for both groups. There were no teeth considered as underfilled. Extruded filling material was completely resorbed in all teeth after a period of 1–6 months. When the effect of filling extents on the success was evaluated, no statistical relationship was found between filling extents and success rates in either of the groups (*p* > 0.05) (Table 1).

**Figure 1:**
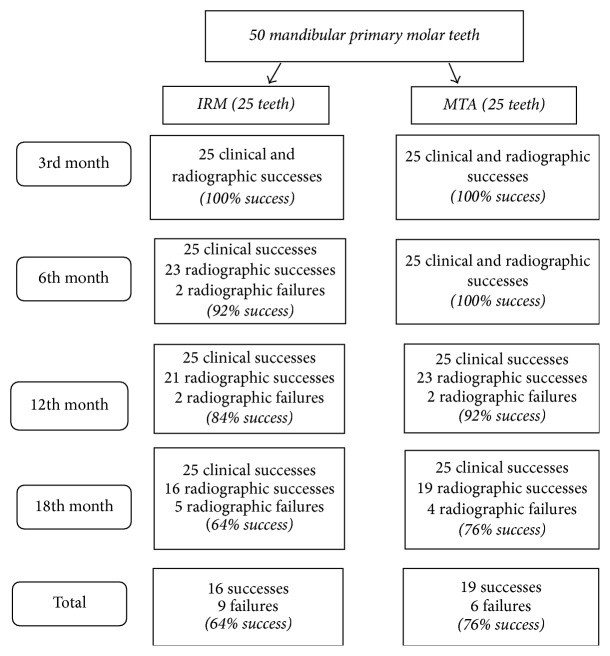
Flowchart showing successes and failures throughout the study.

**Figure 2:**
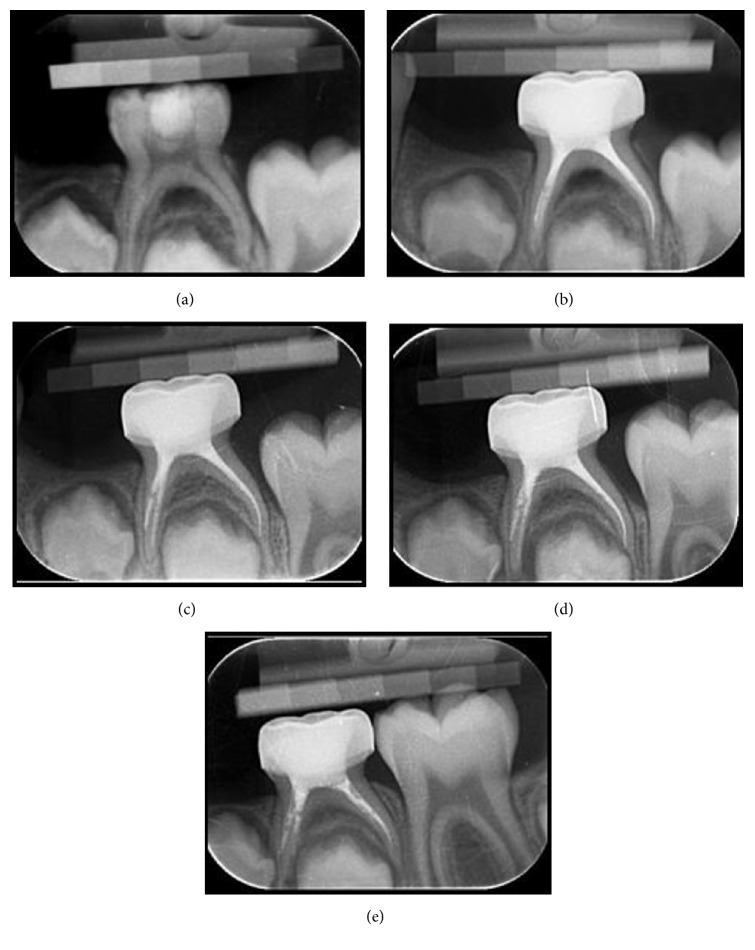
(a) Radiograph of second primary mandibular molar with a lesion in the interradicular area in IRM group. (b) Radiograph of the tooth after treatment. (c) Radiograph of the tooth at the 3rd month visit. (d) Radiograph of the tooth at the 12th month visit. (e) Radiograph of the tooth at the 18th month visit.

**Figure 3:**
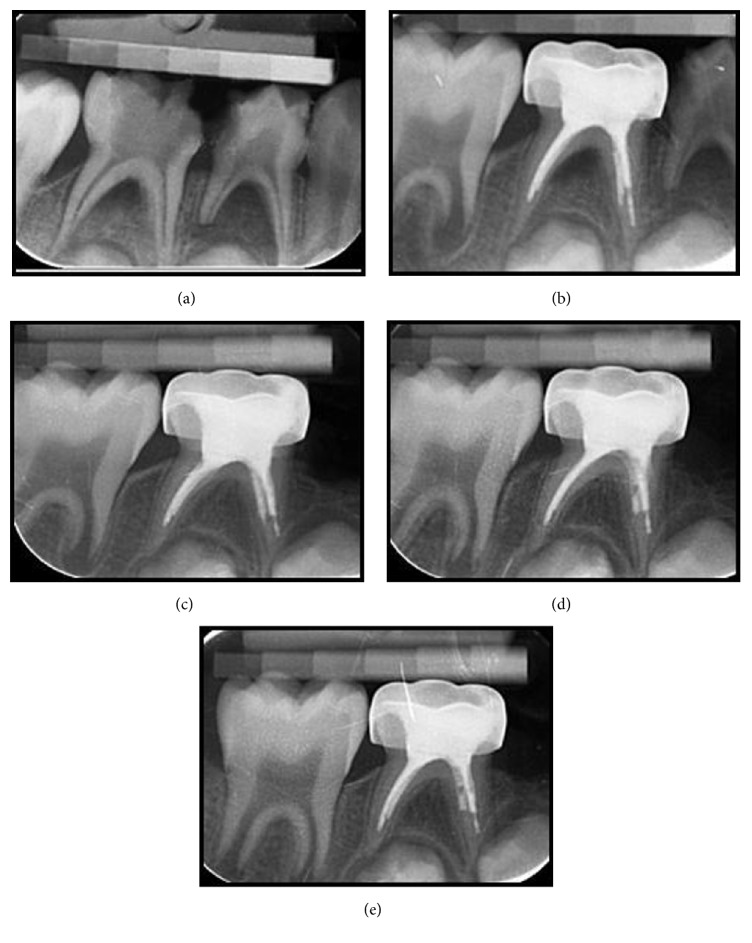
(a) Radiograph of second primary mandibular molar with a lesion in the interradicular area in MTA group. (b) Radiograph of the tooth after treatment. (c) Radiograph of the tooth at the 3rd month visit. (d) Radiograph of the tooth at the 12th month visit. (e) Radiograph of the tooth at the 18th month visit.

At the end of 18 months, for 9 teeth in IRM group and 6 teeth in MTA group, pulpectomies were considered as failures radiographically. Neither of the teeth showed clinical signs or symptoms; therefore there were no clinical failures (Figure 1). Representative successful cases from each group can be seen in Figures 2 and 3. The statistical analysis showed no significant difference between two groups regarding treatment success (*p* > 0.05) (Table 2). Also there were no statistical differences between the success rates at different follow-up appointments (*p* > 0.0025). When the successful cases were evaluated regarding healing time, the statistical analysis revealed that the total healing of the lesions was significantly faster in the MTA group when compared to IRM (*p* < 0.05) (Table 3).

## 4. Discussion

Furcation lesions are more common in primary teeth than in permanent teeth and this phenomenon is attributed to the high permeability of the primary tooth pulpal floor which is a result of the high number of accessory canals, wide dentin tubules, and thin pulpal floors [3–5]. These lesions are important because of their potential hazardous effects on the successor teeth in the neighborhood. In addition to leading to furcation lesions, permeability of the pulpal floor can also cause failure in endodontic treatment by allowing the leakage of bacteria and their toxins into the pulpal space following endodontic treatment [21]. This hypothesis is supported by low success rates of root canal treatments in teeth with furcation lesions [11, 22] and thus the coating agent used on pulpal floor may affect the success of the endodontic treatment in these cases.

A search in literature shows that there are only 2 studies investigating the effect of different applications on the permeability of primary molar pulpal floors. In their in vitro study, Lopes-Silva and Lage-Marques [21] investigated the effect of Er:YAG laser and 2-octyl cyanoacrylate on the permeability and none of the materials completely prevented the permeability. In a similar in vitro study, Guglielmi et al. [4] compared Nd:YAG laser and a self-etch adhesive system (Adhese). They reported that Nd:YAG laser decreased but could not completely eliminate permeability on the pulpal floor. There are no in vivo studies about the subject yet. Therefore, the present study aimed to evaluate the effect of MTA on the root canal treatment success of primary teeth with furcation lesions when used as a base material on the pulpal floor by comparing it to IRM, which is a commonly used material for the coating of pulpal floor following root canal treatment.

According to the results of our study, success rates were 64% for IRM and 76% for MTA group at the end of 18 months. The lowest success rate reported in studies evaluating root canal treatments using Ca(OH)_2_/iodoform is 56% [11] while the highest success rate is 100% [12, 23, 24]. Differences in success rates in different studies are probably a result of differences in follow-up periods, success criteria, and pathological conditions of teeth before treatment. The success rates in our study for both groups are coherent with the literature, closer to the lower bound. This result can be expected since all teeth included in the study had wide lesions in their furcation area and presence and extent of periradicular infection is reported to affect treatment outcome in both permanent and primary teeth [13, 23, 25, 26]. Similar to our study, Nakornchai et al. [11] reported a 56% success rate for primary teeth at the end of 12 months. They used a Ca(OH)_2_/iodoform paste for root canal fillings and most of the teeth included in the study had lesions in the furcation area. However the authors did not limit lesion size while including teeth in their study and this is the possible reason for the lower success rate in their study when compared to our results. For primary tooth pulpectomies with another Ca(OH)_2_/iodoform paste (Vitapex), Trairatvorakul and Chunlasikaiwan [24] also reported a similar success rate (89%) to our study at the end of 12 months for 27 primary molar teeth of which 19 had furcation lesions.

In the present study, the usage of MTA on the pulpal floor was expected to increase success rate in primary molars with furcation lesions since the superior sealing ability of MTA was indicated by many studies and it was also reported that MTA promotes healing in hard tissues [17–20]. MTA was reported to preserve its high pH for a long time owing to calcium release and calcium hydroxide formation [27] and it was reported that MTA shows antimicrobial effect on various microorganisms including* E. faecalis* and* C. albicans* [28–30]. However, although the success rate was higher in the MTA group when compared to IRM group, the difference was not found statistically significant. IRM it is not as biocompatible as MTA and has poorer sealing ability when compared to MTA; thus the success of this material can be explained with its antibacterial affect which was previously reported [31, 32]. Also there are various factors affecting the success of root canal treatment in primary teeth. The adaptation of the filling materials on root canal walls is one of these factors. A perfect sealing in the root canal system cannot be achieved in primary teeth, since cones and condensation are not used during root canal fillings and unlike permanent teeth; the adaptation between root canal walls and filling paste becomes poor [33–35]. As a result even if a perfect sealing could be obtained on the pulpal floor, the leakage in the apical part of the canals may have led to failure. On the other hand, another finding in the present study was that, in the successful cases, lesions healed faster in MTA group when compared to IRM group. Faster healing in MTA group can be attributed to the materials sealing ability, high pH, and hard tissue healing stimulation as well as its antimicrobial effect [17–20, 27–30]. In addition, canal wall adaptation of this material was reported to be better than Portland cement, IRM, LC GIC, Super EBA, and amalgam [15, 16, 36]. Thus, MTA may have decreased healing time by preventing the leakage of microorganisms and their products between pulpal space and lesion area.

## 5. Conclusion

According to the results of the present study, although using MTA did not affect overall healing success, it decreased healing time in primary teeth with furcation lesions.

## Figures and Tables

**Table 1:** Comparison of different filling extents in terms of success in two groups.

	Success	Fail	*p* value
*IRM group *			
Flush-filled	11 (68.8%)	4 (44.4%)	0.397
Overfilled	5 (31.2%)	5 (55.6%)	
*MTA group *			
Flush-filled	13 (68.4%)	2 (33.3%)	0.175
Overfilled	6 (31.6%)	4 (66.7%)	

**Table 2:** Comparison of two groups regarding treatment success during follow-up period.

Controls	IRM group (*n* = 25)	MTA group (*n* = 25)	*p* value
1st month	25/25 (100%)	25/25 (100%)	—
3rd month	25/25 (100%)	25/25 (100%)	—
6th month	23/25 (92%)	25/25 (100%)	0.490
12th month	21/25 (84%)	23/25 (92%)	0.667
18th month	16/25 (64%)	19/25 (76%)	0.355

**Table 3:** Comparison of two groups in terms of lesion healing times in successful cases.

Successful cases	Total healing at the 3rd month	Total healing at the 6th month	*p*
IRM Group	11	5	0,013
MTA Group	19	0	
